# Supplementary figures and images for: The Cul3 ubiquitin ligase engages Insomniac as an adaptor to impact sleep and synaptic homeostasis
Source: PLoS Genet. 2025 Jan 22;21(1):e1011574. doi: 10.1371/journal.pgen.1011574 (PMC11790235; doi:10.1371/journal.pgen.1011574)

**A**

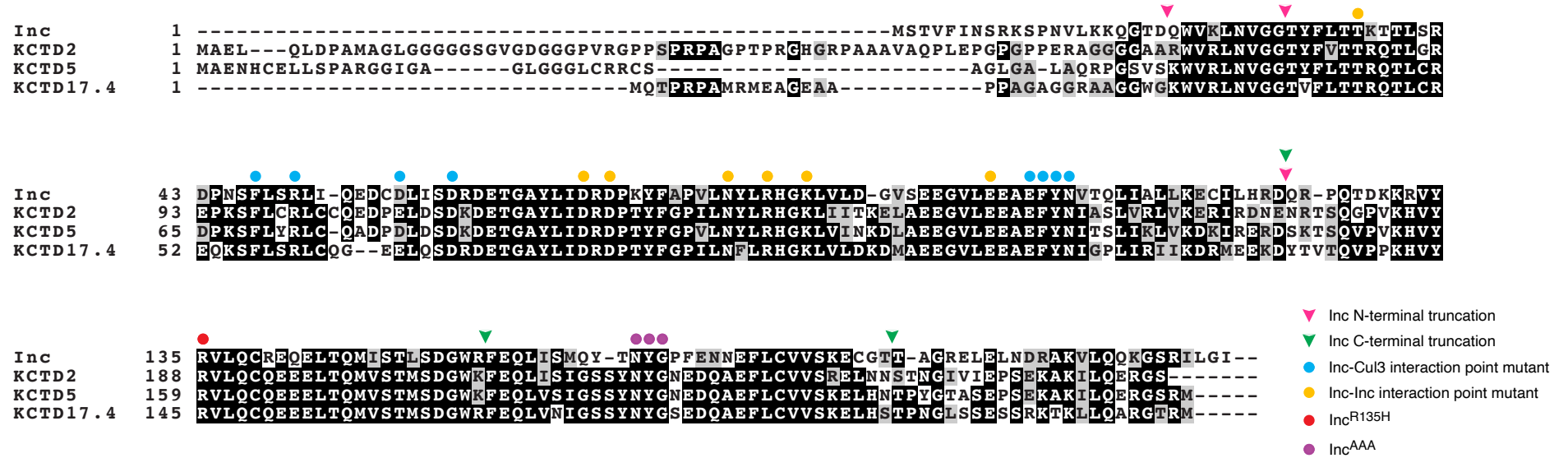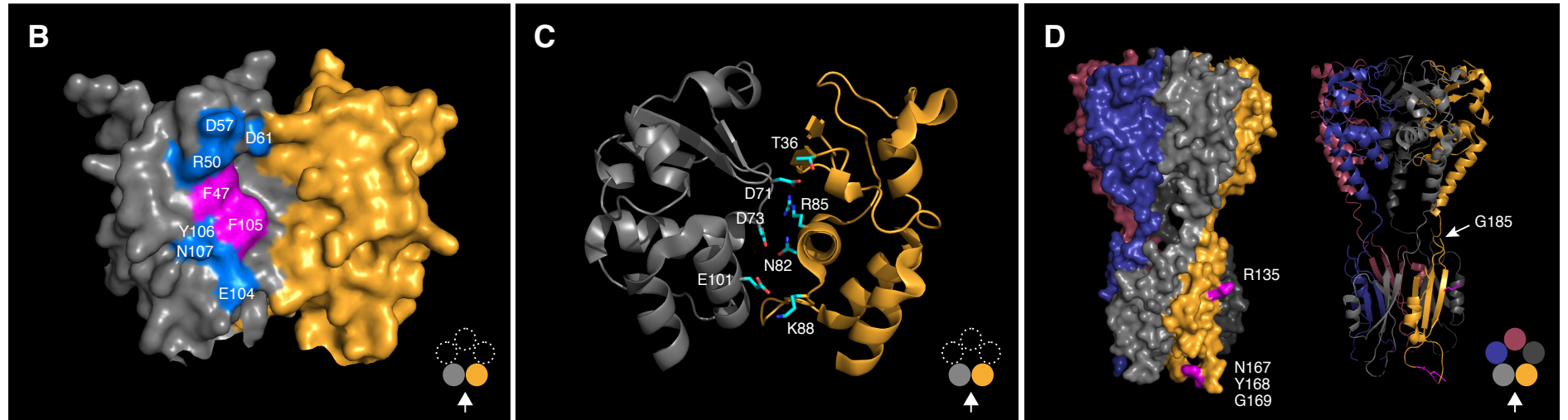

Supplementary Figure 1

Supplement: S1 Fig — (A) Alignment of Inc and its human orthologs. Identical and similar residues are shaded in black and gray respectively. Locations of Inc truncations and point mutants are indicated by arrowheads and closed circles, respectively. (B) Surface rendering of adjacent BTB domains from the crystal structure of human KCTD5 [39]. Here and in subsequent figure panels, Inc residue numbers are superimposed on conserved equivalents in KCTD5. Inc residues whose mutation specifically weakens Inc-Cul3 associations are highlighted in magenta; other mutated residues are highlighted in blue. (C) Ribbon rendering of adjacent KCTD5 BTB domains. Side chains are shown for Inc residues mutated to assess Inc multimerization. Note that the T36A/D71A/R85E triple mutant alters both faces of Inc. (D) Surface (left) and ribbon (right) rendering of human KCTD5. Inc C-terminal point mutants are labeled and indicated in magenta; side chains are shown in the ribbon rendering. Note that KCTD5 residues equivalent to Inc amino acids 186–211 are not resolved in the crystal structure, suggesting that they are disordered. (PDF) [file pgen.1011574.s001.pdf]

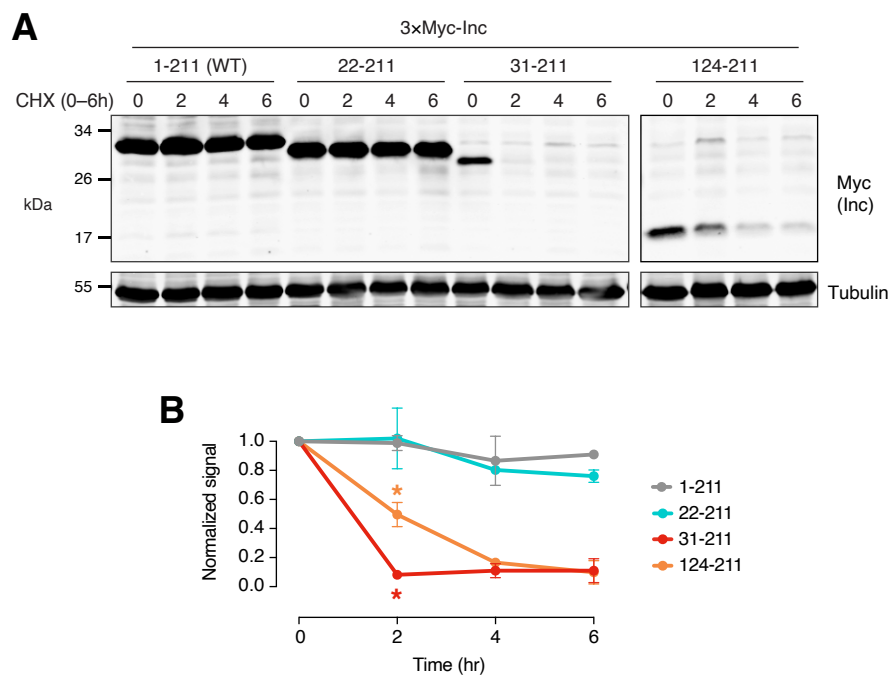

Supplementary Figure 2

Supplement: S2 Fig — (A) Immunoblot of lysates prepared from transiently transfected S2 cells treated with cycloheximide (CHX) for indicated durations. (B) Quantitation of two biological replicates. For all timepoints, Welch’s ANOVA p<0.05 and Dunnett’s tests; *p < 0.05 for comparison to wild-type Inc (1–211). (PDF) [file pgen.1011574.s002.pdf]

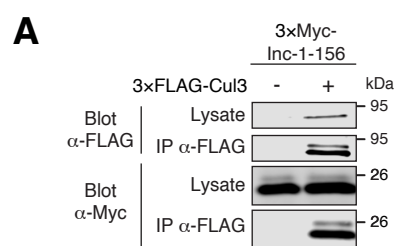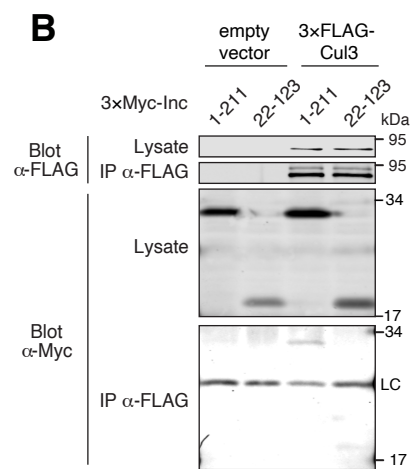

Supplementary Figure 3

Supplement: S3 Fig — (A-B) Co-immunoprecipitation of 3×Myc-tagged Inc1-156 (A) or Inc22-123 (B) with 3×FLAG-Cul3 from transiently transfected S2 cells. (PDF) [file pgen.1011574.s003.pdf]

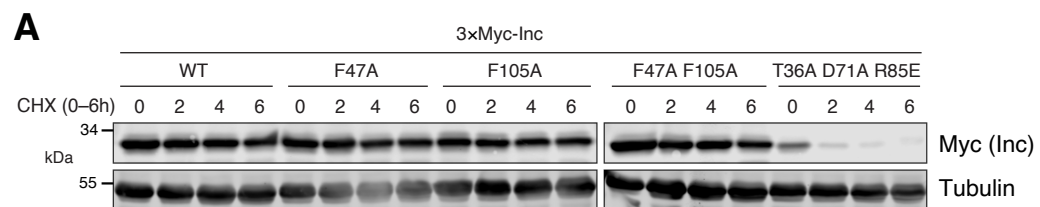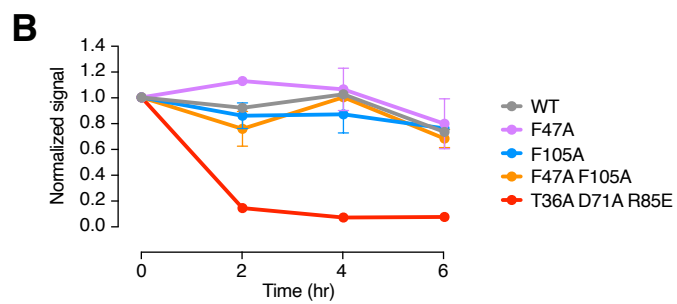

Supplementary Figure 4

Supplement: S4 Fig — (A) Immunoblot of lysates prepared from transiently transfected S2 cells treated with cycloheximide (CHX) for indicated durations. (B) Quantitation of two biological replicates. For all timepoints, Welch’s ANOVA p < 0.05 and Dunnett’s tests for comparison to wild-type Inc. (PDF) [file pgen.1011574.s004.pdf]

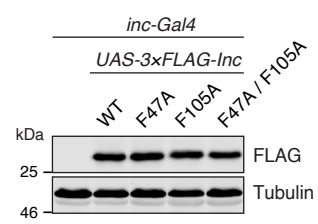

Supplementary Figure 5

Supplement: S5 Fig — Immunoblot of male whole animal lysates expressing 3×FLAG-tagged Inc and Inc point mutants under inc-Gal4 control. Animals are heterozygous for UAS transgenes. (PDF) [file pgen.1011574.s005.pdf]

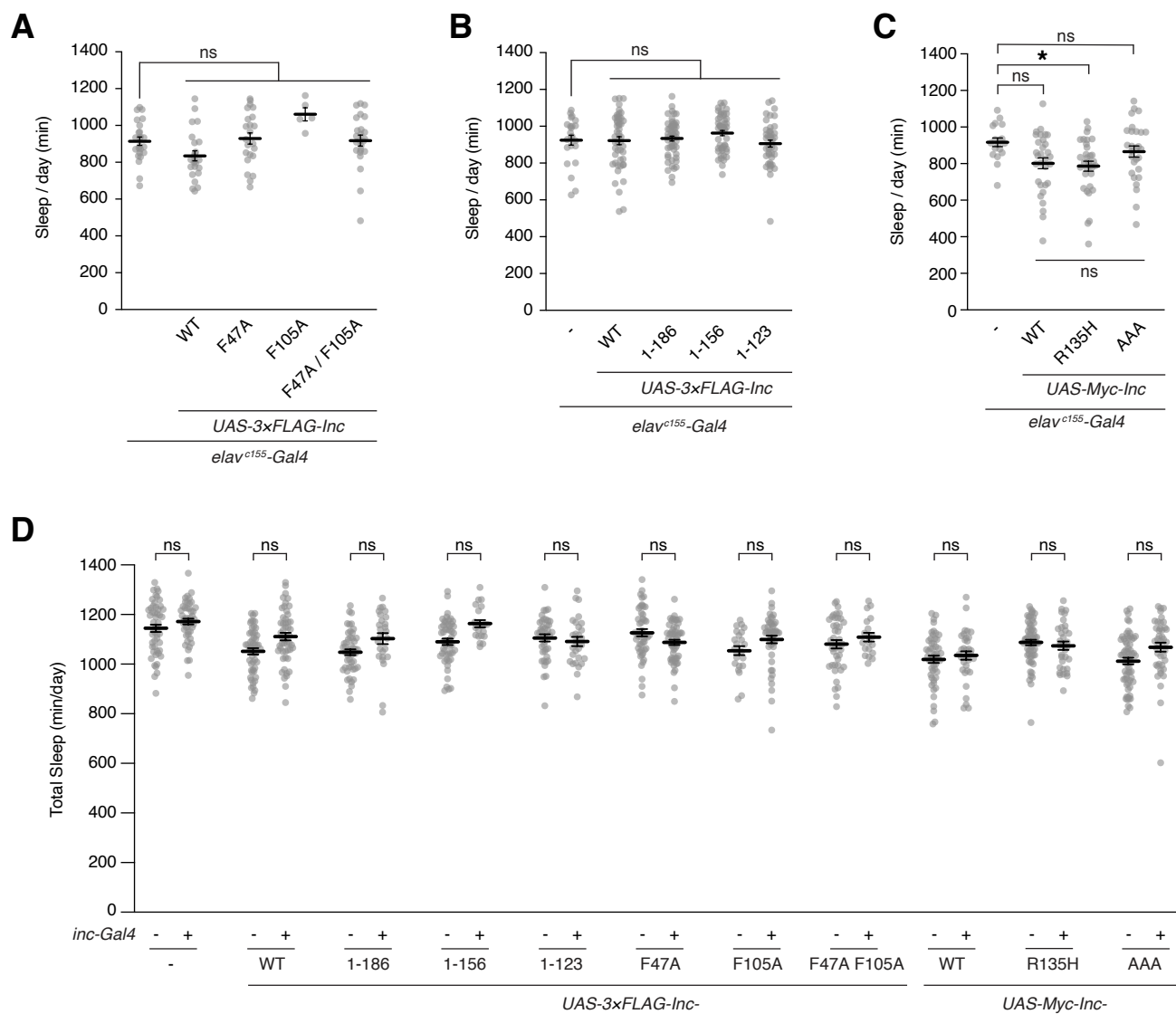

Supplementary Figure 6

Supplement: S6 Fig — Total daily sleep for animals expressing tagged Inc or Inc point mutants under the control of elavc155-Gal4 (A-C) or inc-Gal4 (D). (A) elavc155-Gal4 expression of 3×FLAG-tagged Inc point mutants. n = 5–24. (B) elavc155-Gal4 expression of 3×FLAG-tagged Inc truncations. n = 23–54. (C) elavc155-Gal4 expression of Myc-tagged Inc point mutants. Note that IncR135H is statistically indistinguishable from Inc and IncAAA. n = 18–32. (D) inc-Gal4 expression of 3×FLAG-tagged Myc-tagged Inc point mutants. n = 19–63; For all panels, mean ± SEM is shown. Kruskal-Wallis and Dunn’s tests; *p < 0.01; ns, not significant (p > 0.05). For all panels, animals are heterozygous for UAS transgenes. (PDF) [file pgen.1011574.s006.pdf]

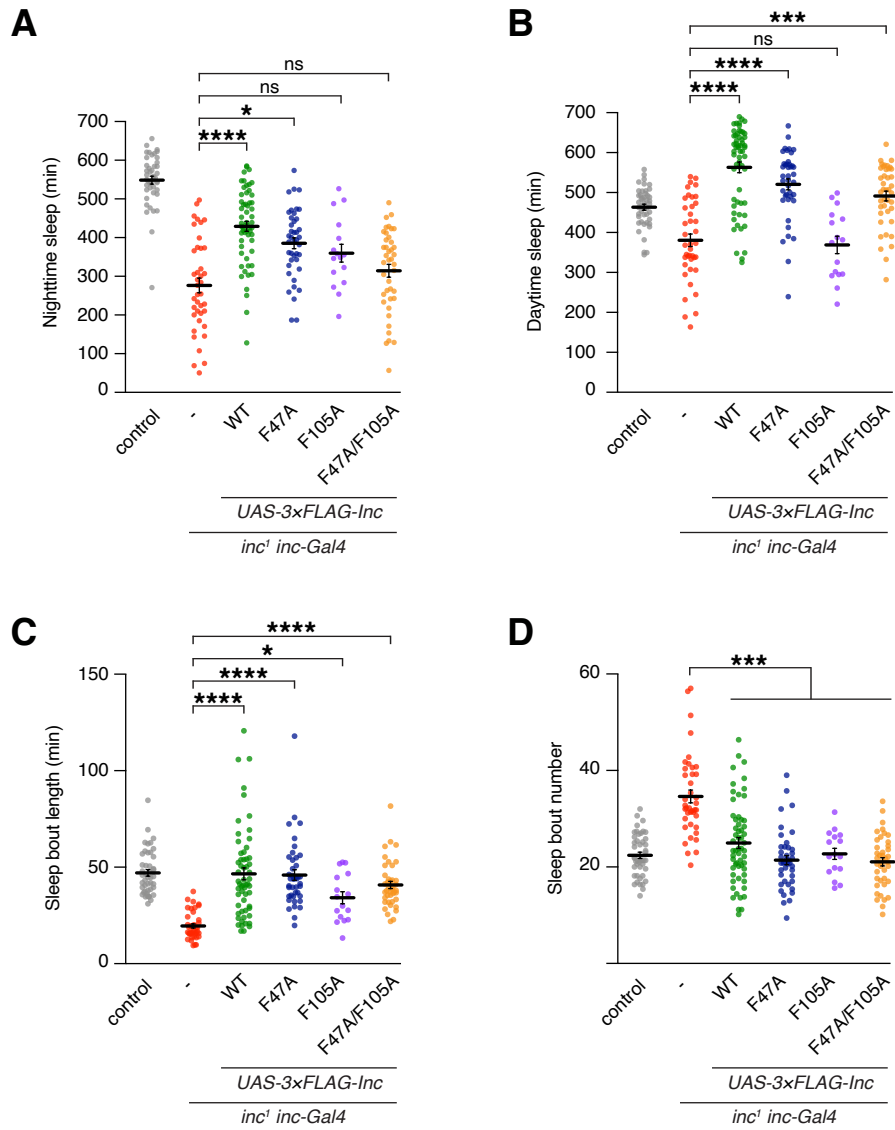

Supplementary Figure 7

Supplement: S7 Fig — (A-D) Sleep parameters for inc1 inc-Gal4 animals expressing 3×FLAG-tagged Inc or Inc point mutants. (A) Nighttime sleep. (B) Daytime sleep. (C) Sleep bout duration. (D) Sleep bout number. Mean ± SEM is shown. n = 16–58 as in Fig 3B; Kruskal-Wallis p < 0.0001 and Dunn’s tests; *p < 0.05; ***p < 0.001; ****p < 0.0001; ns, not significant (p > 0.05). For all panels, animals are heterozygous for UAS transgenes. (PDF) [file pgen.1011574.s007.pdf]

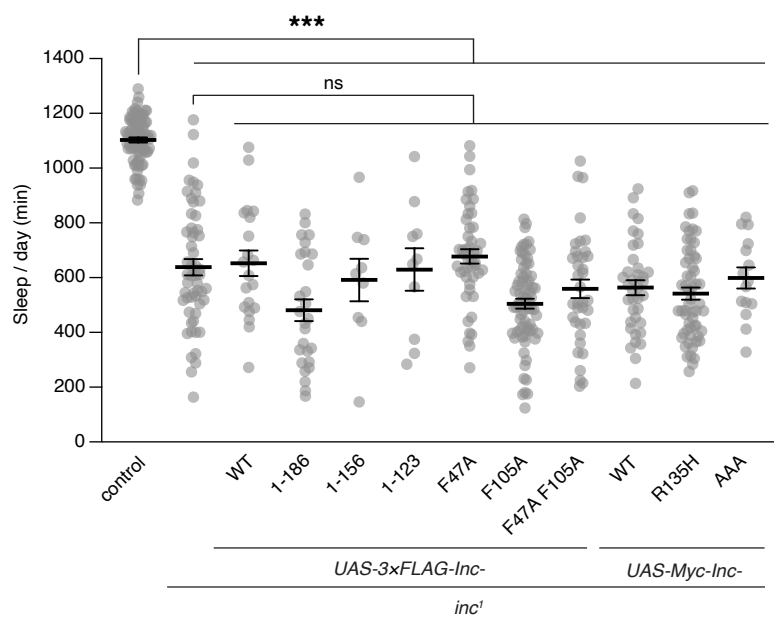

Supplementary Figure 8

Supplement: S8 Fig — Total daily sleep is shown for control, inc1, and inc1 animals heterozygous for indicated UAS transgenes. Mean ± SEM is shown. n = 83–65; Kruskal-Wallis and Dunn’s tests; ***p < 0.001; ns, not significant (p > 0.05). (PDF) [file pgen.1011574.s008.pdf]

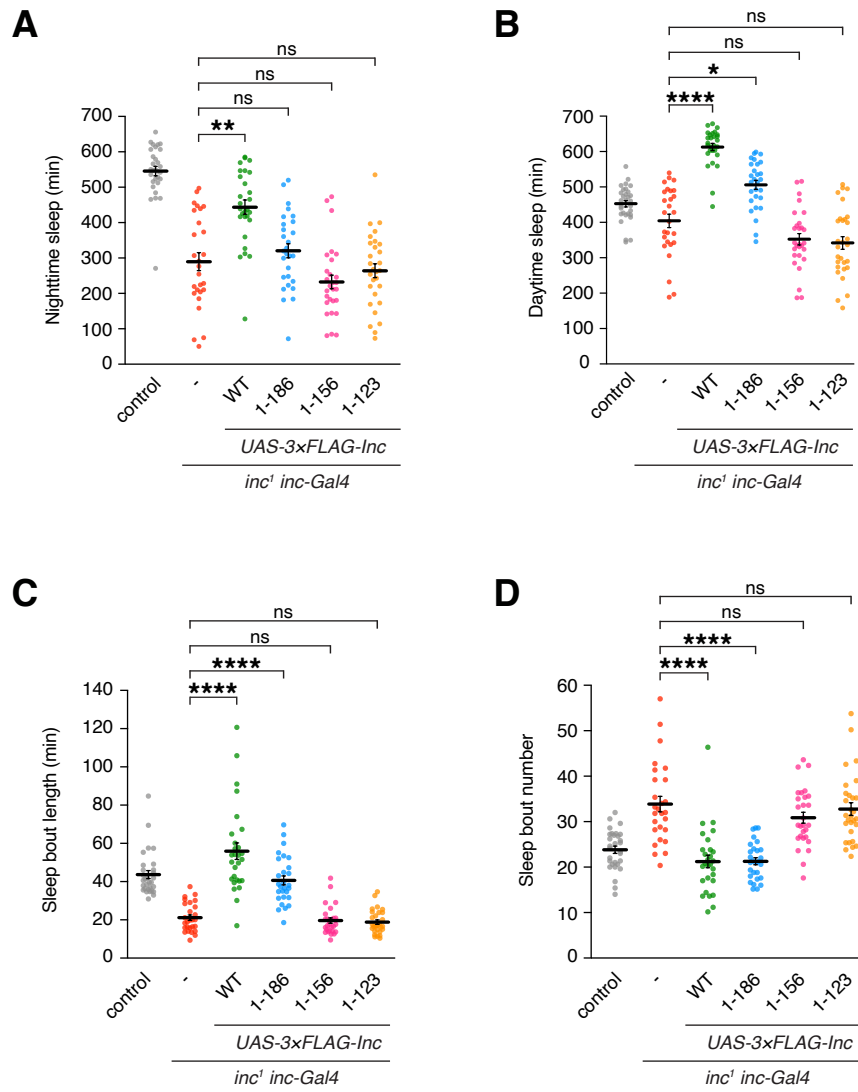

Supplementary Figure 9

Supplement: S9 Fig — (A-D) Sleep parameters for inc1 inc-Gal4 animals expressing 3×FLAG-tagged Inc or C-terminally truncated Inc mutants. (A) Nighttime sleep. (B) Daytime sleep. (C) Sleep bout duration. (D) Sleep bout number. Mean ± SEM is shown. n = 27–30 as in Fig 4B; Kruskal-Wallis p < 0.0001 and Dunn’s tests; *p < 0.05; **p < 0.01; ****p < 0.0001; ns, not significant (p > 0.05). For all panels, animals are heterozygous for UAS transgenes. (PDF) [file pgen.1011574.s009.pdf]

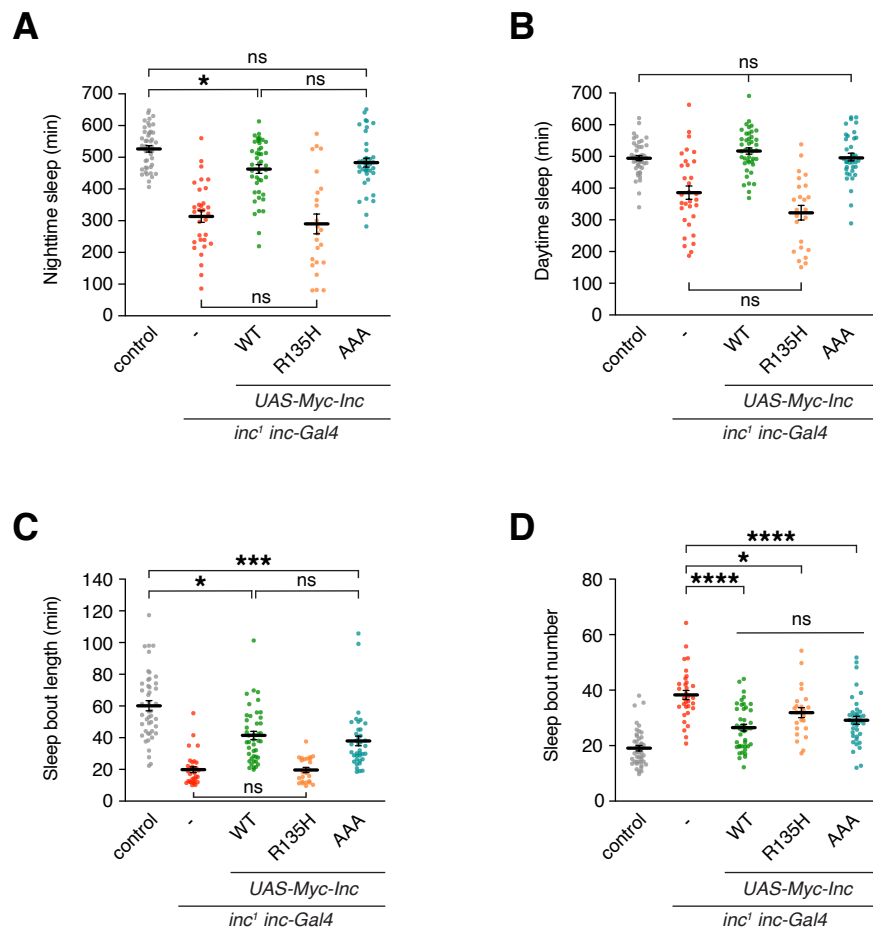

Supplementary Figure 10

Supplement: S10 Fig — (A-D) Sleep parameters for inc1 inc-Gal4 animals expressing 3×Myc-tagged Inc or Inc point mutants. (A) Nighttime sleep. (B) Daytime sleep. (C) Sleep bout duration. (D) Sleep bout number. Mean ± SEM is shown. n = 24–45 as in Fig 6E; Kruskal-Wallis p<0.0001 and Dunn’s tests; *p < 0.05; ***p < 0.001; ****p < 0.0001; ns, not significant (p > 0.05). For all panels, animals are heterozygous for UAS transgenes. (PDF) [file pgen.1011574.s010.pdf]
