# Supplementary material for: The Cul3 ubiquitin ligase engages Insomniac as an adaptor to impact sleep and synaptic homeostasis
Source: PLoS Genet. 2025 Jan 22;21(1):e1011574. doi: 10.1371/journal.pgen.1011574 (PMC11790235; doi:10.1371/journal.pgen.1011574)
Supplement: S1 Table — (PDF) [file pgen.1011574.s011.pdf]

S1 Table. Summary of Inc point mutants targeting Inc-Cul3 interactions

Inc mutant	Inc-Inc binding	Inc-Cul3 binding	Inc stability
F47A	nc	strongly reduced	nc
R50E	reduced	reduced	nc
D57A	nc	nc	nc
D61A	nc	nc	nc
E104K	nc	nc	nc
F105A	nc	strongly reduced	nc
Y106F	nc	nc	nc
N107A	nc	nc	nc
F47A F105A	nc	strongly reduced	nc

nc, no change
