# Supplementary material for: The Cul3 ubiquitin ligase engages Insomniac as an adaptor to impact sleep and synaptic homeostasis
Source: PLoS Genet. 2025 Jan 22;21(1):e1011574. doi: 10.1371/journal.pgen.1011574 (PMC11790235; doi:10.1371/journal.pgen.1011574)
Supplement: S2 Table — (PDF) [file pgen.1011574.s012.pdf]

S2 Table. Summary of Inc point mutants targeting Inc-Inc interactions

Inc mutant	Inc-Inc binding	Inc-Cul3 binding	Inc stability
T36A	nc	nc	nc
T36A D71A	nc	nc	nc
D71A R85E	N/A	N/A	strongly reduced
D73A N82A	nc	nc	nc
K88D E101K	nc	reduced	nc
T36A D71A R85E	N/A	N/A	strongly reduced

nc, no change

N/A, not able to be assessed due to instability
